# The efficacy of the submucosal injection of lidocaine during endoscopic submucosal dissection for colorectal neoplasms: a multicenter randomized controlled study

**DOI:** 10.1007/s00464-020-08017-1

**Published:** 2020-09-28

**Authors:** Masami Ijiri, Takahiro Sasaki, Mikihiro Fujiya, Takuya Iwama, Yuki Murakami, Keitaro Takahashi, Kazuyuki Tanaka, Katsuyoshi Ando, Nobuhiro Ueno, Shin Kashima, Kentaro Moriichi, Hiroki Tanabe, Yusuke Saito, Toshikatsu Okumura

**Affiliations:** 1 Division of Gastroenterology and Hematology/Oncology, Department of Medicine, Asahikawa Medical University, 2-1-1-1, Midorigaoka, Asahikawa, Hokkaido 078-8510 Japan; 2 Department of Gastroenterology, Asahikawa City Hospital, Asahikawa, Hokkaido Japan; 3 Department of Gastroenterology, Asahikawa Kosei Hospital, Asahikawa, Hokkaido Japan

## Abstract

**Background:**

Endoscopic submucosal dissection (ESD) is currently a common procedure although it requires a long procedural time. We conducted a prospective study to determine the efficacy and safety of lidocaine injection for shortening the procedural time and relieving bowel peristalsis during ESD.

**Methods:**

A multicenter randomized controlled study was conducted in three hospitals. Ninety-one patients who underwent colorectal ESD were enrolled. Patients were randomly divided into two groups using the envelope method: the lidocaine group and saline group. The primary endpoint was the procedural time, and the secondary endpoints were the procedural time in each part of the colon and the grade of bowel peristalsis and the incidence and amounts of antispasmodic drugs use and adverse events.

**Results:**

The patients’ demographics were not markedly different between the two groups. The mean procedural time in the lidocaine group was not markedly different from that in the saline group. In contrast, at the proximal site, the procedural time in the lidocaine group (57 min) was significantly shorter in the saline group (80 min). The grade of bowel peristalsis in the lidocaine group (0.67) was significantly lower than in the saline group (1.17). Antispasmodic drug use was significantly rarer in the lidocaine group than in the saline group. The incidence of adverse events was not markedly different between the two groups.

**Conclusions:**

Local lidocaine injection is a feasible option for preventing bowel peristalsis, particularly in the proximal colon, leading to a reduced procedural time for ESD and decreased antispasmodic drug use.

University Hospital Medical Information Network Center (UMIN number: 000022843).

Endoscopic submucosal dissection (ESD) is a common procedure for the treatment of colorectal T1 cancer, particularly large lesions. However, ESD carries a high risk of perforation and requires a longer procedural time than other endoscopic procedures, including polypectomy and endoscopic mucosal resection [1]. Submucosal fibrosis, difficulties maintaining scope positioning, and bowel peristalsis are thought to underlie the long procedural time [2–4].

Several new cutting devices have been developed to easily cut through submucosal fibrosis [5, 6], and pocket-creation methods [7] have been used to maintain a good endoscopic view. However, few procedures for controlling bowel peristalsis have been developed. Anticholinergic drugs, such as butylscopolamine, are frequently used to relieve bowel peristalsis, although these drugs have some adverse effects, including tachycardia, an increased intraocular pressure, and dipsesis. Glucagon is used as a substitute of butylscopolamine in patients with cardiac diseases or glaucoma, although that drug carries risks of inducing hyperglycemia and late-onset hypoglycemia [8]. For these reasons, the administrable amounts of these drugs are limited, particularly in elderly patients or patients with such comorbidities.

Lidocaine is a local analgesic that has long been in use, and its safety in appropriate amounts has been established. The safety of local lidocaine injection during ESD in patients with gastric cancer has recently been proposed [9], and the efficacy of lidocaine spraying during colonoscopy has also been reported [10]. However, the efficacy and safety of lidocaine injection during ESD in patients with colorectal cancer has not yet been explored.

We conducted a multicenter randomized control study to determine the efficacy and safety of lidocaine injection during ESD for the treatment of colorectal T1 cancer.

## Methods

### Study design and ethical considerations

This is a multicenter randomized non-blinded control study that was approved by the Ethics Committees of Asahikawa Medical University (15089-3) and other participating institutes and registered with the University Hospital Medical Information Network Center (UMIN number: 000022843). Written informed consent was obtained from all patients enrolled.

### Participants

From November 2015 to March 2019, 91 patients diagnosed with colorectal adenomas, T1 cancers, or neuroendocrine tumors that were indicated for ESD were enrolled in this study. The exclusion criteria were as follows: patients with severe cardiac disease, severe renal disease, severe cardiac infectious disease, severe diabetes, severe dehydration or malnutrition, and hemorrhagic diathesis as well as those in whom participation was deemed difficult by physician and those < 16 years old.

### Randomization

The envelope method was used for randomization. Before ESD, patients were divided into two groups at a 1:1 ratio: the lidocaine group and the saline (placebo) group.

### Procedures

Colonoscopy was started with sedation of 5–10 mg midazolam with no scopolamine butylbromide and/or glucagon. During the insertion of the colonoscope, 20 mg scopolamine butylbromide and/or 1 mg glucagon were injected only when the endoscopist called for an injection. Glucagon was used for patients with heart disease, prostate hypertrophy, and glaucoma as well as those ≥ 75 years old, and scopolamine butylbromide was used for all other patients.

On reaching the lesion, 1% lidocaine or saline was injected at both the oral and anal sides of the lesion before starting ESD. After starting ESD, 1% lidocaine or saline was injected every 15 min. A maximum of 20 ml of 1% lidocaine or saline was injected. If ESD could not be successfully completed after injecting ≥ 20 ml of 1% lidocaine or saline, then the procedure was performed without the injection of 1% lidocaine or saline. Scopolamine butylbromide and/or glucagon were injected when the endoscopist considered antispasmodic drugs necessary.

The grade of bowel peristalsis was classified as follows: score 0, no peristalsis; score 1, less peristalsis with no influence for ESD; score 2, mild peristalsis with influence for ESD; score 3, severe peristalsis in which ESD was able to be continued with some treatment.

### Endpoints

The primary endpoint was the procedural time of ESD. The secondary endpoints were the bowel peristalsis score, amounts of antispasmodic drugs used, safety of lidocaine injection, and procedural time in each location. A subgroup analysis was performed to analyze the differences according to the location (proximal colon which included the cecum and ascending colon or distal colon).

### Statistical analyses

The IBM SPSS statistics software program, version 25, was used for the statistical analysis. A *t*-test or Mann–Whitney *U*-test was used for the analysis of continuous variables. The Chi-square test or Fisher’s exact test was for nominal variables. *P* values of less than 0.05 were judged to indicate significance.

## Results

### Demographics of enrolled patients

**Fig. 1:**
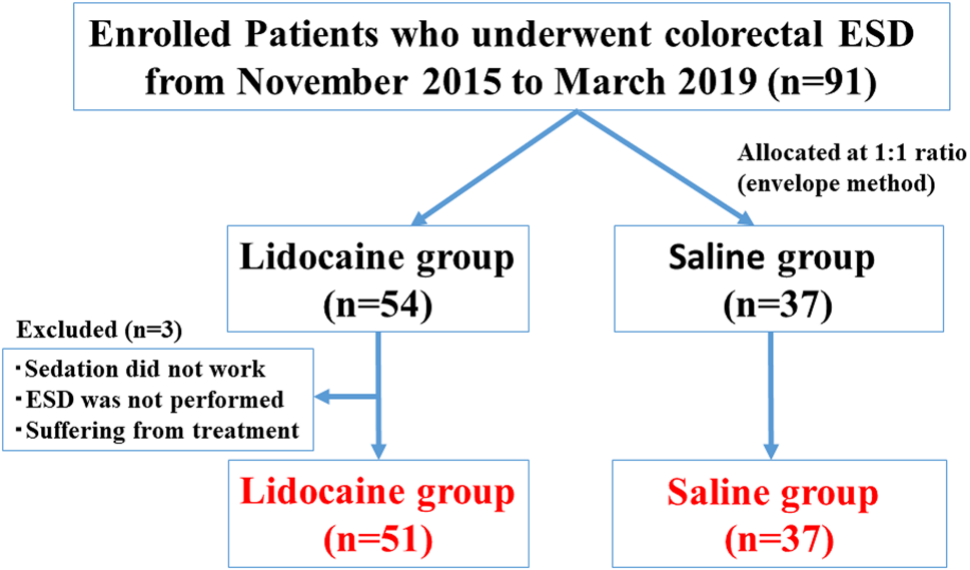
Flowchart of the patients enrolled in this study

Ninety-one patients were enrolled in this prospective study. These patients were randomly divided into the lidocaine group (*n* = 54) and saline group (*n* = 37) using the envelop method. One patient in whom sedation did not work and 2 in whom ESD was not completed due to severe fibrosis were excluded from this study, leaving 51 patients in the lidocaine group and 37 in the saline group for the analysis (Fig. 1). The age, gender, comorbidities, lesion location, histological type, invasion depth, tumor size, and grade of histological fibrosis were not markedly different between the lidocaine and saline groups (Table 1).

**Table 1:** Patients’ demographics

	Lidocaine group (*n* = 51)	Saline group (*n* = 37)	*P* value
Sex (male: female)	30:21:00	19:18	*P* = 0.32
Age (mean ± SD)	69.2 ± 11.9	70.8 ± 8.2	*P* = 0.60
Comorbidities	20 (39%)	14 (38%)	*P* = 0.54
Location			
Proximal colon:distal colon	19:32	17:20	*P* = 0.27
Histological type			
Adenoma:carcinoma:NET	30:19:02	22:14:01	*P* = 0.95
Invasion depth			
M:SM	15:06	9:05	*P* = 0.47
Tumor size(mm)	20 (20, 30)	25 (20, 40)	*P* = 0.39
Proximal colon	25 (20, 30)	30 (20, 42.5)	*P* = 0.10
Distal colon	22.5 (13.75, 30)	20 (20, 30)	*P* = 0.62
Median(Q1, Q3)			
Fibrosis	9 (18%)	5 (14%)	*P* = 0.41

### Primary endpoints

**Fig. 2:**
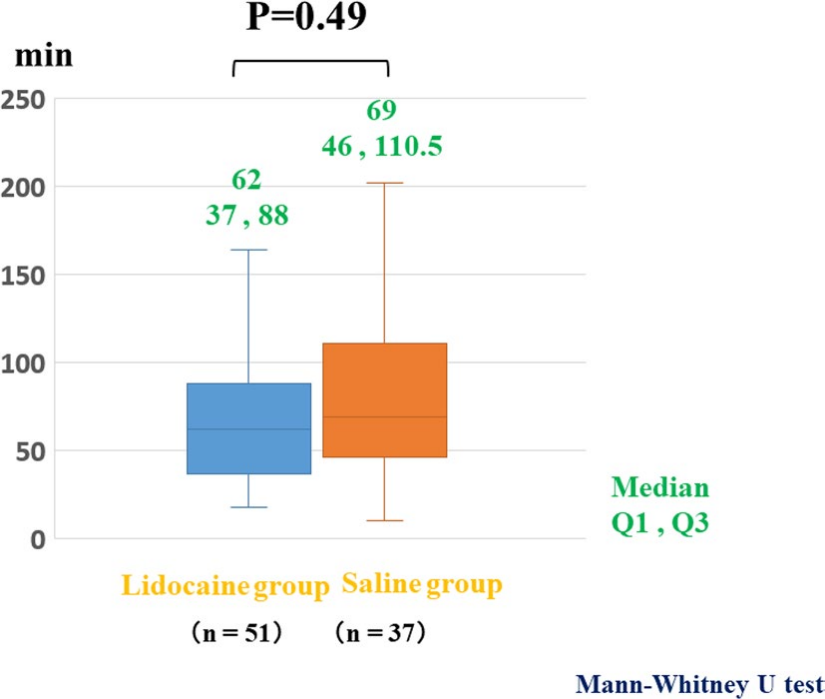
Procedural times in the lidocaine and saline groups in the whole colon. The procedural time in the lidocaine group (62 min) was not significantly different from that in the saline group (69 min) (*p* = 0.49)

The median procedural time in the lidocaine group (62 min) was similar to that in the saline group (69 min). No significant difference in the procedural time was observed between the groups (Fig. 2).

### Secondary endpoints

**Fig. 3:**
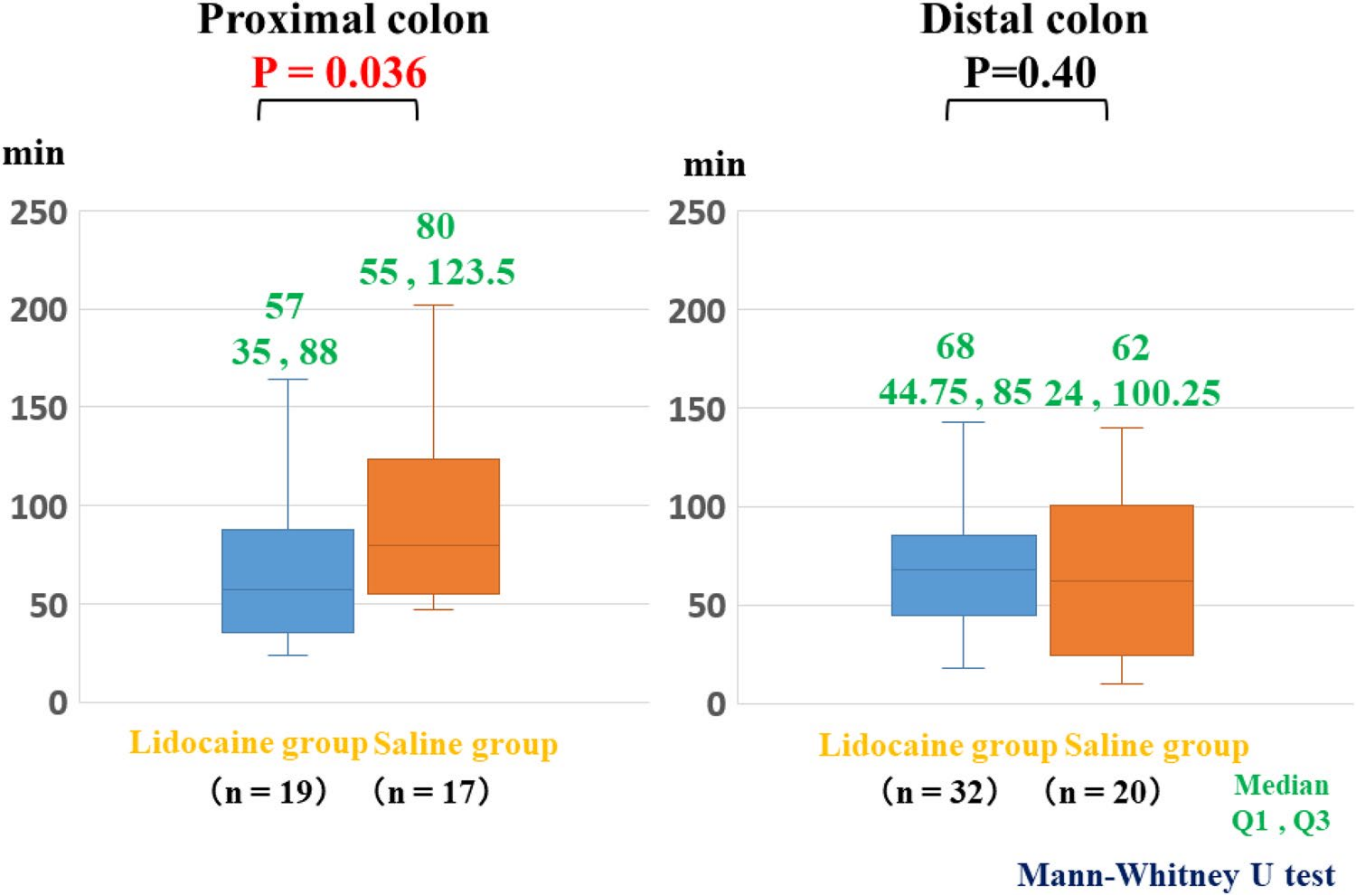
Procedural times at the proximal and distal sites of the colon. In the proximal colon, the mean procedural times in the lidocaine and saline groups were 57 and 80 min, respectively. The time in the lidocaine group was significantly shorter than in the saline group (*p* < 0.05). In the distal colon, there was no marked difference between the groups

**Fig. 4:**
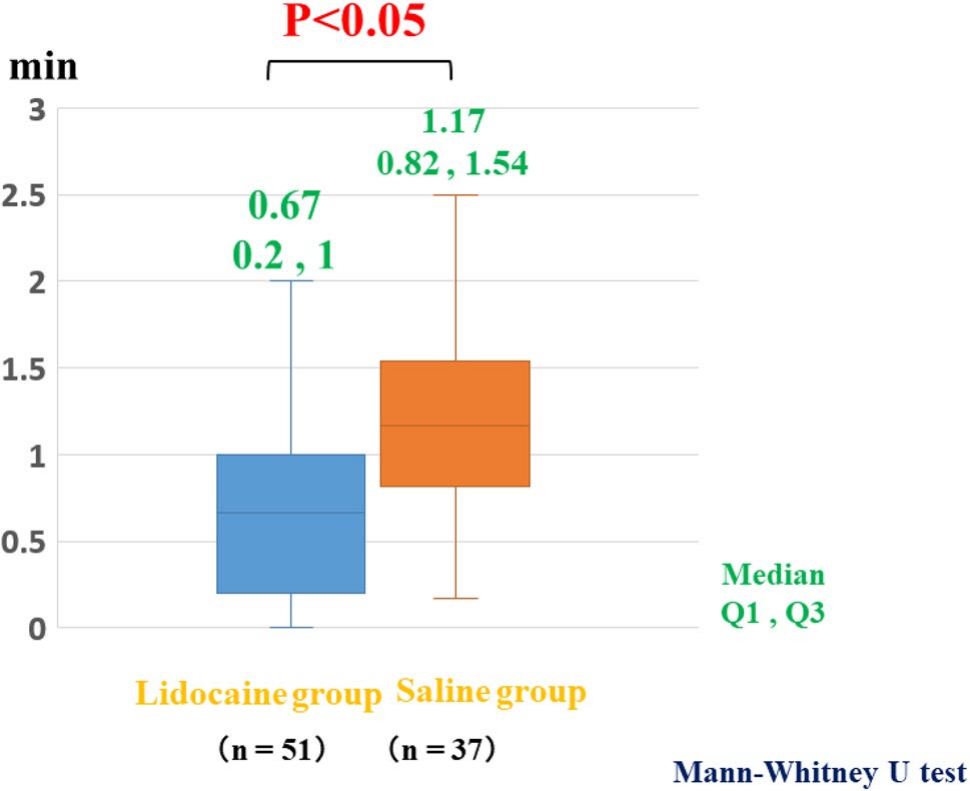
Bowel peristalsis scores in the lidocaine and saline groups. The mean bowel peristalsis scores in the lidocaine and saline groups were 0.67 and 1.17, respectively. The score was significantly higher in the lidocaine group than in the saline group (*p* < 0.05)

**Fig. 5:**
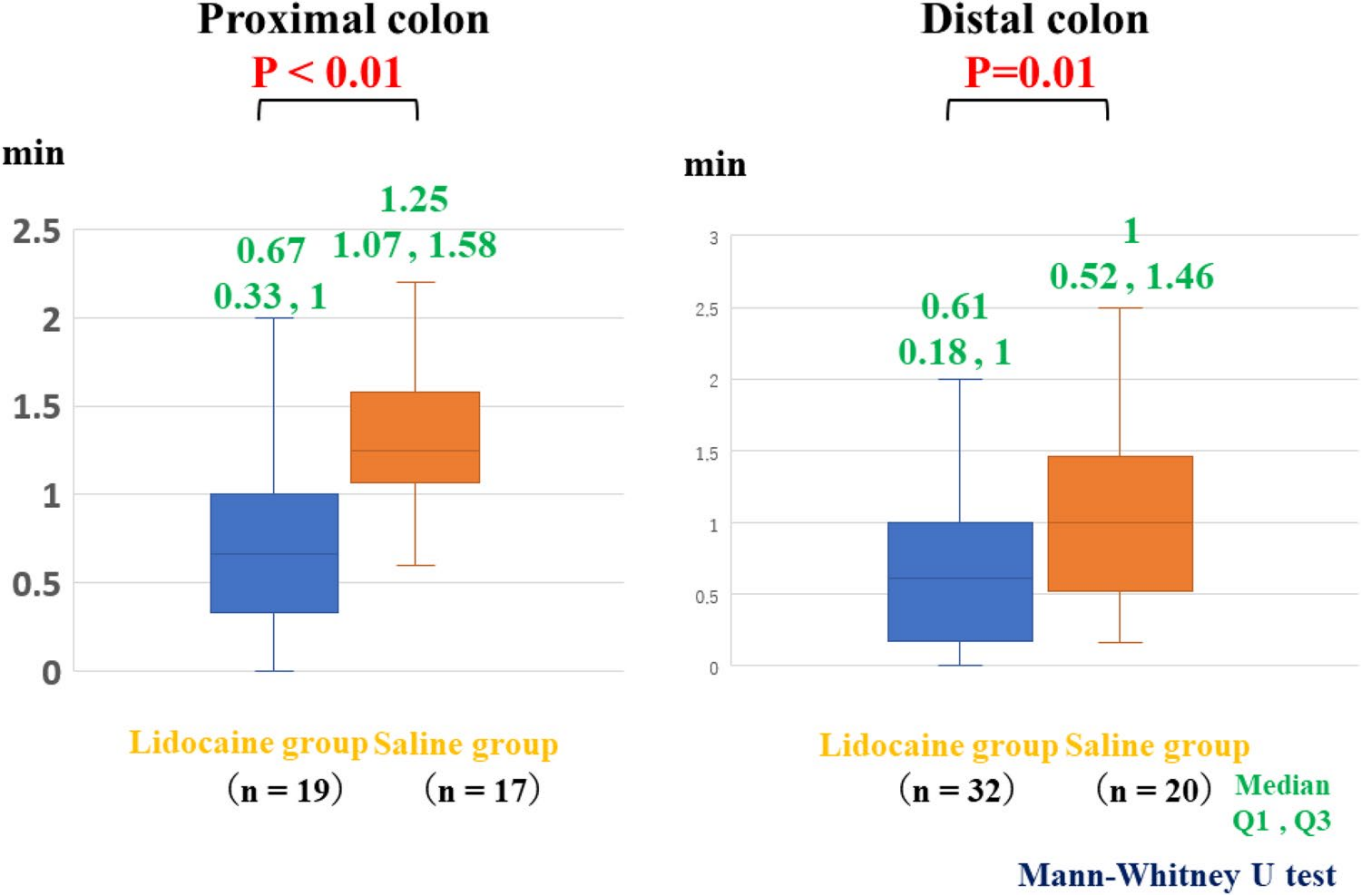
Bowel peristalsis scores at the proximal and distal sites of the colon. In the proximal colon, the mean bowel peristalsis scores in the lidocaine and saline groups were 0.67 and 1.25, respectively. The bowel peristalsis scores in the lidocaine group were significantly lower than in the saline group (*p* < 0.01). In the distal colon, the mean bowel peristalsis scores in the lidocaine and saline groups were 0.61 and 1.00, respectively. The bowel peristalsis scores in the lidocaine group were significantly lower than in the saline group (*p* = 0.01)

The median procedural time in the lidocaine group for removing tumors in the proximal colon (57 min), which included the cecum and ascending colon, was significantly shorter than that in the saline group (*p* = 0.05), while no marked differences in the procedural time were observed between the groups in the distal colon, which included the transverse colon, descending colon, sigmoid colon, and rectum (Fig. 3). The bowel peristalsis score of the lidocaine group (0.67) was significantly lower than that of the saline group (*p* < 0.05) (Fig. 4). The median bowel peristalsis score of the lidocaine group in the proximal colon was significantly lower than that in the saline group (*p* < 0.01), and the median bowel peristalsis score in the distal colon of the lidocaine group was significantly lower than that in the distal colon of the saline group (*p* = 0.01) (Fig. 5). The rate of using additional antispasmodic drugs in the lidocaine group (6%) was significantly lower than that in the saline group (35%) (*p* < 0.05) (Table 2). The incidence of adverse events, including perforation, penetration, bleeding, hypotension, bradycardia, tachycardia, and hypoxemia, was similar between the groups (Table 3).

**Table 2:** Amount and incidence of antispasmodic drug use

	Lidocaine group (*n* = 51)	Saline group (*n* = 37)	*P* value
Total volume (ml)	8 (6, 12)	10 (6, 15)	*P* = 0.17
Cases using additional antispasmodic agents	*n* = 3 (6%)	*n* = 13 (35%)	*P* = 0.001
Butyl scopolamine bromide	*n* = 1	*n* = 4	
Glucagon	*n* = 2	*n* = 9

**Table 3:** Adverse events

	Lidocaine group (*n* = 51)	Saline group (*n* = 37)	*P* value
Minor perforation*	4 (8%)	1 (3%)	*P* = 0.30
Perforation**	0 (0%)	2 (5%)	*P* = 0.17
Bleeding	2 (4%)	2 (5%)	*P* = 0.56
Hypotension	1 (2%)	1 (3%)	*P* = 0.67
Tachycardia	1 (2%)	0 (0%)	*P* = 0.58
Bradycardia	0 (0%)	2 (5%)	*P* = 0.17
Decrease of SpO2	1 (2%)	0 (0%)	*P* = 0.58

*Cases with suspected perforation during ESD without any symptoms associated with the perforation

**Cases with perforation with obvious clinical symptom associated with the perforation

## Discussion

While the safety of local lidocaine injection in gastric ESD has been proposed [5], the present prospective study investigated the efficacy and safety of local lidocaine injection in colorectal tumors for the first time, illustrating that local lidocaine injection prevented bowel peristalsis during ESD and shortened the procedural time in the proximal colon. The study also showed the high safety of local lidocaine injection, suggesting the usefulness of local lidocaine injection for efficient and safe ESD.

Submucosal fibrosis, difficulties maintaining the scope position, and bowel peristalsis are known to underlie the long procedural time of ESD [2–4]. A number of new cutting devices have been developed for easily cutting submucosal fibrosis. In addition, traction devices, such as the S–O clip [5], as well as the balloon overtube-guided technique [6] and pocket-creation methods [7] have been used to maintain a good endoscopic view. However, few procedures for controlling bowel peristalsis have been developed. While spraying lidocaine has been proposed as a potential procedure for controlling bowel peristalsis, there is no established procedure that can be applied for the minutes-long slowing of peristalsis. The present study proposed for the first time the feasibility of a local lidocaine injection when applied for minutes-long slowing of peristalsis. The combination of new cutting devices, traction devices, and local lidocaine injection may be suitable when performing ESD in difficult cases with large-sized colorectal tumors, submucosal fibrosis, and/or a poor endoscopic view.

ESD at the hepatic and splenic flexures has been considered difficult [11, 12]. Operating an endoscope at the proximal colon has been recognized as more difficult in comparison to the distal colon due to the presence of numerous high semilunar folds and the long scope insertion. In the present study, the procedural time in the proximal colon in the lidocaine group was significantly shorter than that in the saline group, while the procedural time in the distal colon was not markedly different between the two groups, suggesting that relief of the bowel peristalsis might contribute to the shortening of the procedural time in the proximal colon. Based on the results, local lidocaine injection is thought to be recommended for removing colorectal tumors, particularly those in the proximal colon. Antispasmodic drug use was significantly rarer in the lidocaine group than in the saline group, although the procedural time was not markedly different between the two groups. When avoiding antispasmodic drug use during ESD in high-risk cases, such as older patients and/or those with severe comorbidities, local lidocaine injection is recommended even in the distal colon.

Lidocaine blocks the sodium channel and decreases the entry of sodium into neuron cells, thereby inhibiting the activities of both sensory and motor nerves [13]. While it is not clear why lidocaine inhibited bowel peristalsis, we assumed that lidocaine decreased the sensitivity of the mucosal surface, leading to the stimulation of bowel peristalsis by factors in the intestinal lumen. Another possibility was that the submucosal injection of lidocaine directly suppressed the nerve plexus.

Two limitations associated with this study warrant mention. First, this study was blinded for patients but not for endoscopists, which might have influenced the results, including the procedural time and amount of antispasmodic drugs used. Second, the study did not optimize the amount or interval of lidocaine injection. Further double-blinded studies are therefore needed in order to clarify the appropriate amount and interval of lidocaine injection for preventing bowel peristalsis during colorectal ESD.

In conclusion, local lidocaine injection is a feasible option for preventing bowel peristalsis, particularly in the proximal colon, thereby reducing the ESD procedural time and antispasmodic drug use.
